# NSE-Verlauf nach Reanimation – wenn ein Wert nichts wert ist: ein Case-Report

**DOI:** 10.1007/s00115-024-01681-x

**Published:** 2024-06-11

**Authors:** Maike R. Pollmanns, Jule K. Adams, Manuel Dafotakis, Turgay Saritas, Christian Trautwein, Samira Abu Jhaisha, Alexander Koch

**Affiliations:** 1 Klinik für Gastroenterologie, Stoffwechselerkrankungen und internistische Intensivmedizin, Uniklinik RWTH Aachen, Aachen, Deutschland; 2 Klinik für Neurologie, Uniklinik RWTH Aachen, Aachen, Deutschland; 3 Klinik für Nieren- und Hochdruckkrankheiten, rheumatologische und immunologische Erkrankungen, Uniklinik RWTH Aachen, Aachen, Deutschland

## Hintergrund

Mit den Fortschritten in der Intensiv- und Notfallmedizin und Verbesserungen in der rettungsdienstlichen Versorgung überleben mehr Patient:innen einen Herz-Kreislauf-Stillstand [9]. Dabei stellt die Prognoseabschätzung nach einem Herz-Kreislauf-Stillstandes weiterhin eine komplexe Herausforderung dar [7].

Zur Abschätzung der neurologischen Prognose wird unter anderem die Bestimmung des Serumspiegels der neuronenspezifischen Enolase (NSE) empfohlen [4]. Höhere NSE-Werte nach einem Herz-Kreislauf-Stillstand können auf einen Hirnschaden hinweisen. Der Grenzwert, bei dessen Überschreitung man in der aktuell veröffentlichten Literatur von einer eingeschränkten neurologischen Prognose ausgeht, variiert je nach Quelle und liegt zwischen 33 ng/ml [10] und 97 ng/ml [1].

## Kasuistik

Im vorliegenden Bericht wird der Fall einer 22-jährigen Patientin geschildert, die nach einem außerklinischen Herz-Kreislauf-Stillstand unklarer Dauer bei Asphyxie nach Alkoholintoxikation erfolgreich reanimiert wurde. Nach einer initialen Laienreanimation erfolgte eine Weiterbehandlung durch den Rettungsdienst mit Rückkehr eines stabilen Spontankreislaufs (ROSC) nach 25 min.

**Abb. 1:**
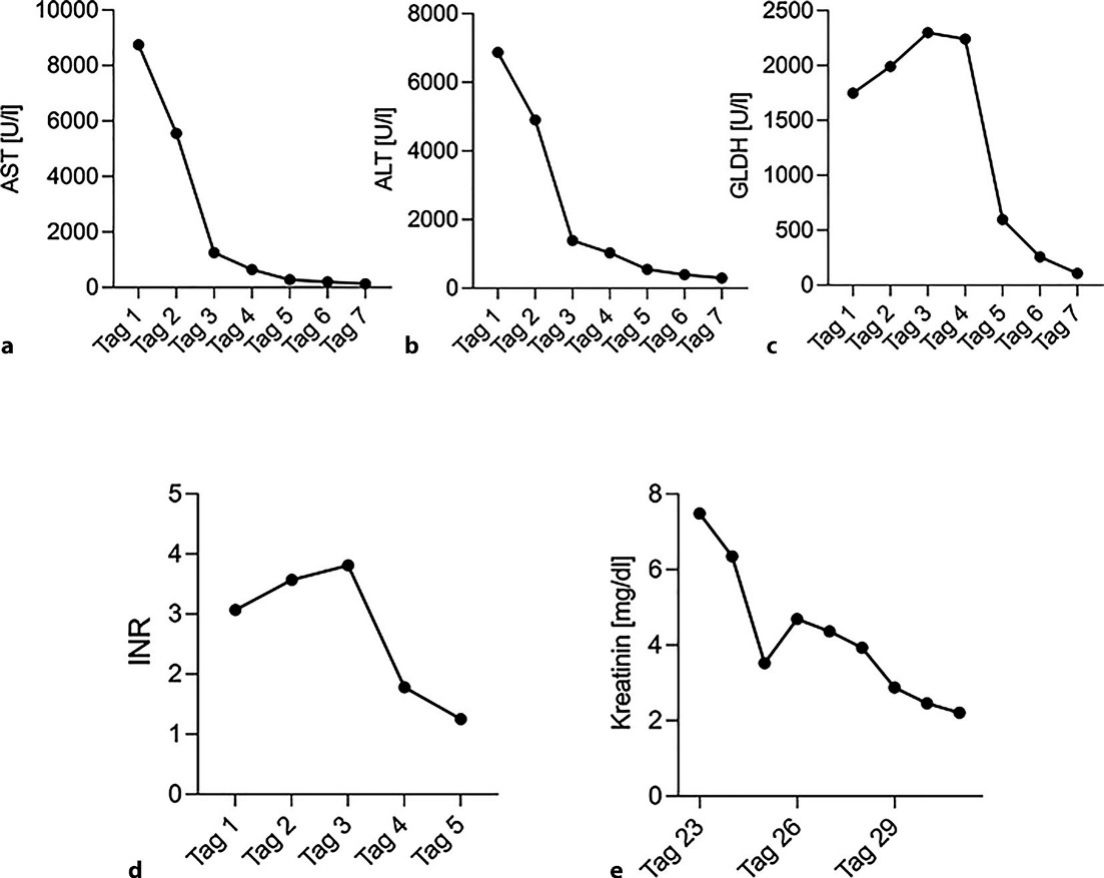
**a** Verlauf AST beginnend am 1. Tag nach CPR; **b** Verlauf ALT beginnend am 1. Tag nach CPR; **c** Verlauf GLDH beginnend am 1. Tag nach CPR; **d** Verlauf INR beginnend am 1. Tag nach CPR; **e** Verlauf Kreatinin beginnend an Tag 23 nach CPR

Bei der Erstaufnahme in einem externen Krankenhaus zeigte ein kraniales Computertomogramm einen Tag nach der Reanimation keine sicheren Auffälligkeiten. Die Patientin wurde unmittelbar nach Aufnahme auf die externe Intensivstation mittels hypothermer Temperaturkontrolle für 22 h gekühlt. Bei Zeichen einer ischämischen Hepatitis mit drohendem Leberversagen (Alanin-Aminotransferase [ALT] maximal 145,75 µmol/sl, Grenzwert 0,583 µmol/sl; Aspartat-Aminotransferase [AST] maximal 114,5 µmol/sl; Grenzwert 0,583 µmol/sl, Glutamat-Dehydrogenase 38,3133.; Grenzwert < 0,083, International Normalized Ratio [INR] maximal 3,57; Abb. 1a–d), erfolgte die Verlegung auf die internistische Intensivstation unserer Klinik. Die Patientin war zu diesem Zeitpunkt mit 0,308 µg/kg KG pro min hochgradig katecholaminpflichtig, tief sediert und intubiert. Die Wertigkeit der klinisch-neurologischen Untersuchung war entsprechend eingeschränkt und letztlich nur der vorhandene Pupillenreflex verwertbar.

**Abb. 2:**
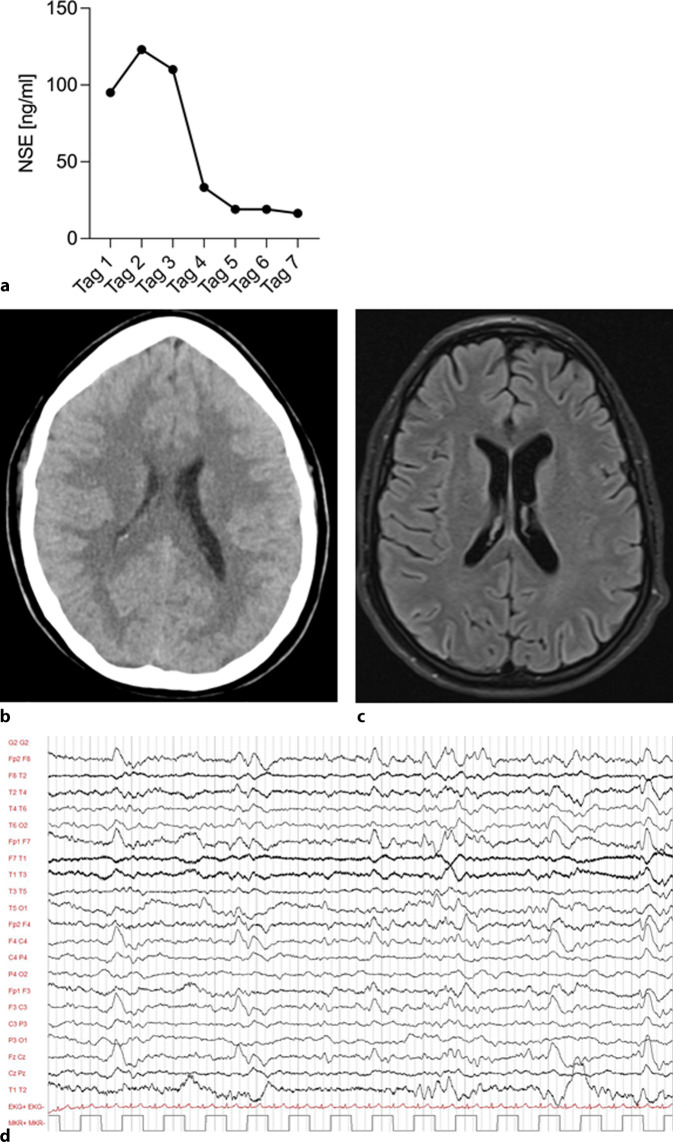
**a** NSE-Verlauf beginnend am 1. Tag nach CPR; **b** kraniales CT an Tag 3 nach CPR; **c** kraniales MRT am 20. Tag nach CPR; **d** EEG am 13. Tag nach CPR

Ein NSE-Wert von 123 ng/ml (Abb. 2a) wurde 48 h nach Reanimation ermittelt, gefolgt von einem weiteren kranialen CT am selben Tag (Abb. 2b). Dieses zeigte eine altersgerechte Hirndarstellung ohne Hinweise auf einen hypoxischen Schaden oder einen erhöhten Hirndruck. Der NSE-Serumspiegel zeigte sich im Verlauf fallend (Abb. 2a). Bei einem akuten Nierenversagen aufgrund eines Crush-Syndroms (Myoglobin max. 206,3429 nmol/l, Grenzwert 3,3143 nmol/l; Kreatininkinase [CK] max. 220,8 µmol/sl, Grenzwert 2,833 µmol/sl) wurde extern eine Nierenersatztherapie mit Hämoadsorption begonnen. Nach 21 Tagen konnte die Dialyse beendet werden. Die Kreatininwerte zeigten sich im Anschluss regredient (Abb. 1e). Der Weaningprozess von der Beatmung gestaltete sich anspruchsvoll, konnte jedoch nach 10 Tagen erfolgreich abgeschlossen werden. Die weiterführende Diagnostik zur Abschätzung der neurologischen Prognose mittels kranialer magnetresonanztomographischer(MRT)-Untersuchung (Abb. 2c), Elektroenzephalogramm (EEG; Abb. 2d) sowie Medianus-SEP (Tab. 1) verblieb ohne relevante Auffälligkeiten.

**Tab. 1:** SEP N. medianus am 5. Tag nach CPR

Durchgang	Beschriftung	N20 (ms)	P25 (ms)	N20-P25 (µV)	N10 (ms)
1	Cortex, Erb	18,91	24,11	0,85	10,16
2	Cortex, Erb	18,39	23,65	0,88	10,05

Das klinische Bild verbesserte sich zunehmend durch physiotherapeutische, ergotherapeutische und logopädische Übungen sowie durch eine L‑Dopa-Therapie. Dies ermöglichte schließlich am Tag 34 nach Reanimation die Verlegung der Patientin in eine neurologische Rehabilitationsklinik. Zum Zeitpunkt der Verlegung fand sich klinisch ein mildes dyston-spastisches Syndrom als Folge der Reanimation.

## Diskussion

Im Rahmen der Kasuistik wird die NSE als Prognosefaktor nach einer kardiopulmonalen Reanimation beleuchtet. Eine NSE-Erhöhung kann neben des Untergangs zerebraler Neurone auch andere Ursachen haben: Erhöhte Serumwerte sind bei Tumoren wie dem kleinzelligen Bronchialkarzinom nachweisbar [3]. Durch die hohe Konzentration in Erythrozyten kommt es außerdem zu einem NSE-Anstieg in Folge von Hämolyse, diese kann in vivo – durch Trauma oder iatrogene Interventionen wie Nierenersatztherapie (RRT; [6]) – oder in vitro vorliegen.

Die Höhe des NSE-Wertes im Serum wird oft als Biomarker für zentralnervöse Schädigung nach kardiopulmonaler Reanimation verwendet. Lange galt ein NSE-Wert im Serum von 33 ng/ml 48 h nach Reanimation als Grenzwert, ab dem man von einer deutlich eingeschränkten neurologischen Prognose ausgeht [10]. Die für diesen Grenzwert zugrunde liegende Studie wurde 2006 veröffentlicht und liegt somit vor der standardisierten Postreanimationsbehandlung und der Einführung der hypothermen Temperaturkontrolle. Die Leitlinie für die weitere Behandlung nach Reanimation der European Resuscitation Council von 2021 gibt einen höheren Wert von über 60 ng/ml als Grenzwert an [4].

Der vorliegende Fall unserer Patientin unterstreicht die Komplexität der Interpretation der NSE-Werte. Hier kommen für die Erhöhung der NSE-Werte mehrere Faktoren als ursächlich in Betracht.

Eine eingeschränkte Nierenfunktion scheint keinen wesentlichen Einfluss auf die Höhe des NSE-Serumspiegels zu haben (Abb. 1e). Eine Korrelation zwischen NSE und Kreatinin konnte in der Vergangenheit nicht gezeigt werden [8].

Eine weitere mögliche Ursache könnte eine erhebliche Hämolyse sein, die durch die Nierenersatztherapie verursacht worden sein könnte [6]. Bei unserer Patientin lagen die Haptoglobinwerte jedoch mit 6,3 µmol/l im Normbereich (Grenzwert 4,9–21,8 µmol/l), was das Vorliegen einer relevanten Hämolyse unwahrscheinlich erscheinen lässt.

Möglicherweise wurde der deutlich erhöhte NSE-Wert unserer Patientin zu Beginn durch die ischämische Hepatitis mit drohendem akutem Leberversagen verursacht (Abb. 1a). Frühere Berichte legen nahe, dass erhöhte NSE-Werte bei Patient:innen mit Zirrhose oder fulminanter Hepatitis auftreten [8]. Dabei könnten nicht nur die durch hepatische Enzephalopathie (HE) verursachten neuronalen Schäden zu den erhöhten Werten beigetragen haben, sondern auch ein reduzierter Abbau in der Leber. Es wurde gezeigt, dass bei Patient:innen nach Hepatektomie die NSE-Werte signifikant postoperativ anstiegen [2]. Demnach scheint eine akute Schädigung der Leber einen Einfluss auf den NSE-Serumwert haben zu können. In Bezug auf den Fall der hier geschilderten Patientin mit hypoxischer Hepatitis nach CPR kann diese Studie nahelegen, dass die bei der Patientin gemessenen NSE-Werte auch durch die Hepatitis erhöht waren.

Die wichtigste Limitation dieser Fallbeschreibung besteht darin, dass es sich hierbei um einen Einzelfallbericht handelt. Die Verallgemeinerung von Erkenntnissen aus einem Einzelfall auf eine breitere Population sollte mit Vorsicht erfolgen.

Dennoch bleibt festzuhalten, dass die frühzeitige Prognoseeinschätzung für die medizinische Versorgung und Betreuung der Patient:innen von zentraler Bedeutung ist, insbesondere wenn ein ungünstiger Verlauf absehbar ist. Die 2023 von der Neurocritical Care Society (NCS) und der Deutschen Gesellschaft für Neurointensivmedizin (DGNI) veröffentlichte Leitlinie zur neurologischen Prognostizierung bei erwachsenen Überlebenden eines Herzstillstandes empfiehlt einen multimodalen Ansatz zur Prognoseeinschätzung [5]. Dazu können verschiedene klinische Anzeichen, wie beispielsweise generalisierte frühzeitige Myoklonien und eine fehlende Pupillenreaktion, sowie apparative Diagnostik, wie EEGs mit Befunden wie Burst-Suppression-Mustern oder isoelektrischen Mustern, der Ausfall der zentralen Komponenten der Medianus-SEP und biochemische Marker wie die NSE, beitragen. Eine umfassende Prognosestellung sollte daher stets auf verschiedenen Indikatoren basieren, um eine fundierte Entscheidungsgrundlage für die weitere Behandlung und Betreuung der Patient:innen zu schaffen.

## Fazit für die Praxis

Zusammenfassend zeigt dieser Fall, dass prognostische Aussagen – auch im Hinblick auf die Fortführung lebenserhaltender Therapien – nicht auf dem Boden einzelner und somit isolierter Parameter getroffen werden sollten, sondern immer im Gesamtkontext verstanden werden müssen.
